# Supplementary figures and images for: Identification and Characterization of Abiotic Stress Responsive CBL-CIPK Family Genes in Medicago
Source: Int J Mol Sci. 2021 Apr 28;22(9):4634. doi: 10.3390/ijms22094634 (PMC8124885; doi:10.3390/ijms22094634)

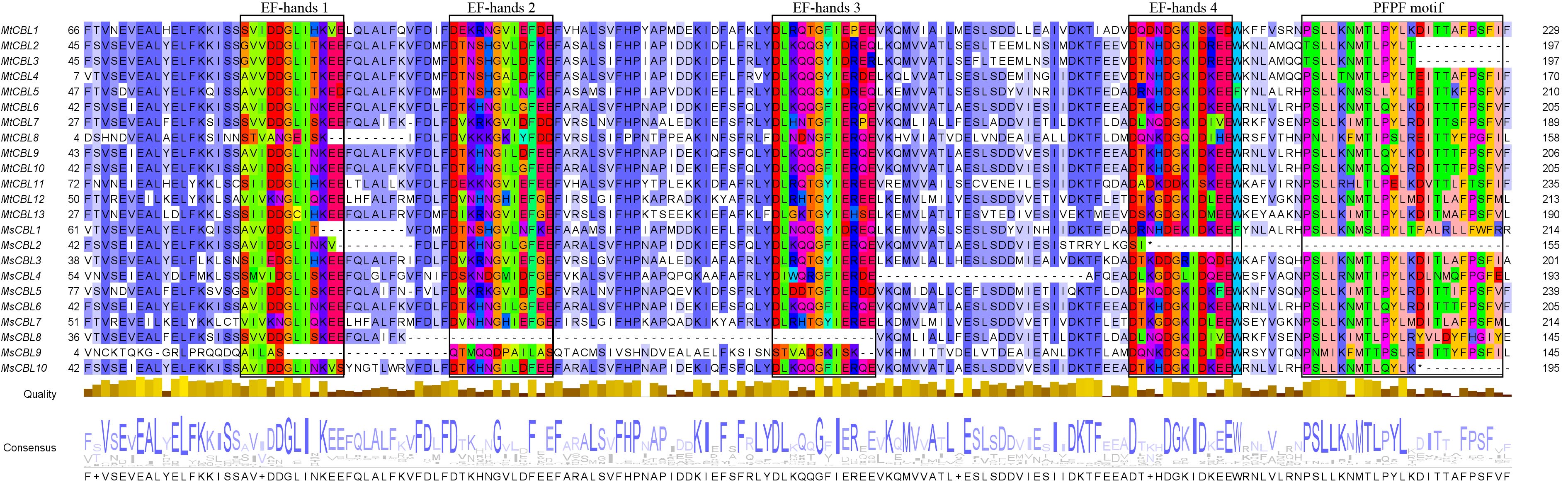

Supplement: Supplementary file 1 [file ijms-22-04634-s001.zip › Supporting Information/Fig S1.jpg]

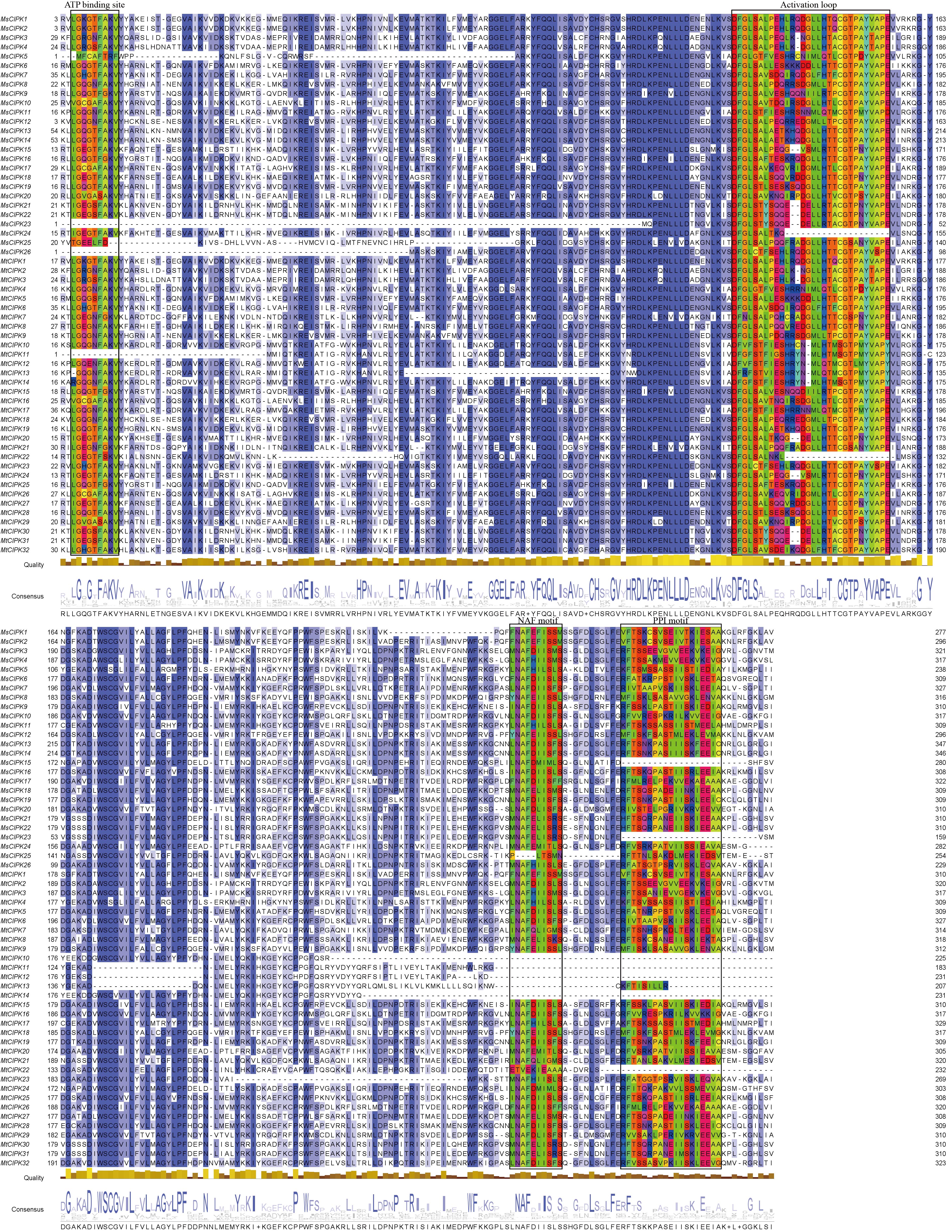

Supplement: Supplementary file 1 [file ijms-22-04634-s001.zip › Supporting Information/Fig S2.jpg]
